# Reduction and outcome of posterior pilon fractures with intercalary fragments: a retrospective cohort study comparing the transfibular and posteromedial approaches

**DOI:** 10.1186/s10195-025-00851-0

**Published:** 2025-05-29

**Authors:** Li Ying, Can Yao, Bin Wang, Junbo Liang, Guofu Chen

**Affiliations:** Department of Orthopaedics, Taizhou Hospital of Zhejiang Province, Affiliated to Wenzhou Medical University, No. 150 Ximen Street, Linhai, 317000 Zhejiang Province China

**Keywords:** Posterior pilon fracture, Fibular fracture, Intercalary fragment, Transfibular fracture region approach, Long-term follow-up

## Abstract

**Background:**

The transfibular fracture region (TFFR) approach can be utilized for managing posterior pilon fractures associated with intercalary fragments. However, its long-term outcomes remain unreported. This study aimed to compare the long-term clinical outcomes of the TFFR approach and the posteromedial approach for posterior pilon fractures (Klammer type 2/3, Danis–Weber type B) associated with displaced intercalary fragments over an average 8 year follow-up.

**Method:**

From 2012 to 2018, a cohort of consecutive patients who underwent open reduction and internal fixation surgery via either the TFFR approach or the posteromedial approach for posterior pilon fracture associated with intercalary fragments were enrolled for this study. Clinical outcomes were evaluated over an average 8 year (range 5–12 years) follow-up. The surgical duration, number of intraoperative fluoroscopies, and postoperative complications were recorded. Functional outcomes were assessed using the Foot and Ankle Outcome Score (FAOS), Foot and Ankle Ability Measure (FAAM), and Short Form-36 (SF-36) score at last follow-up.

**Results:**

Seventy-nine patients were included in the final analysis, including 43 in the TFFR group and 36 in the posteromedial group. No significant differences between the two groups were observed in the FAOS (*p* = 0.679) or its specific components for symptoms (*p* = 0.264), pain (*p* = 0.963), activities of daily living (ADL, *p* = 0.102), sports (*p* = 0.156), or quality of life (*p* = 0.859). There was also no significant difference between the two groups in the FAAM-ADL (*p* = 0.408), FAAM-Sport (*p* = 0.617), and SF-36 scores (*p* = 0.757). Nevertheless, the surgical duration was shorter in the TFFR group (*p* < 0.001).

**Conclusion:**

The TFFR approach is not inferior to the posteromedial approach. For posterior pilon fractures with lateral malleolar fractures in the same plane, the TFFR approach may be preferred owing to its potential to reduce surgical time and the use of a single incision.

*Level of Evidence* Level III, retrospective cohort study.

**Supplementary Information:**

The online version contains supplementary material available at 10.1186/s10195-025-00851-0.

## Introduction

Posterior pilon fracture is a unique variant of ankle fracture that involves the entire posterior tibial lip, including the posterior half of the medial malleolus [13, 25]. Its mechanism of injury differs from that of standard trimalleolar and classical pilon fractures, as it results from a combination of rotational force and low-energy vertical impact [1, 13, 19, 28]. The prognosis of posterior pilon fractures is generally worse, particularly when posteromedial fragments or intercalary fracture fragments are poorly reduced [20, 21, 25]. To improve postoperative ankle function, the treatment of posterior pilon fractures has gained increasing attention over the past two decades. Various surgical approaches are currently used for posterior pilon fractures, including the posterolateral approach, posteromedial approach, and transfibular fracture region (TFFR) approach [1, 4, 6, 9, 11, 13, 15, 24]. However, the optimal treatment remains controversial.

The posterolateral approach is commonly used, as it allows surgeons to treat posterior malleolar and distal fibular fractures through a single incision [13]. However, intercalary fracture fragments cannot be directly visualized or reduced. The posterior bone block may be too large to turn over, as it is restricted by the posterior tibiofibular ligament [10]. The “open book fashion” technique described by Klammer et al. facilitates joint surface visualization but requires more extensive soft tissue dissection on the posterior side and may still necessitate an additional posteromedial incision [13]. The posteromedial approach provides direct access to the posterior pilon joint surface and facilitates direct reduction of intercalary fragments, leading to satisfactory outcomes [9]. However, the posteromedial approach is unable to treat lateral malleolar fractures through the same incision and may cause more soft tissue complications [27]. As most posterior pilon fractures are combined with distal fibular fractures, both of which are located in the same plane, we have utilized a TFFR approach for the treatment of posterior pilon fractures with intercalary fragments since 2012 [6]. This feasible approach enables surgeons to treat posterior pilon, lateral malleolar fracture, and intercalary fragments through a single posterolateral incision. The short-term clinical outcomes at an average 2 year follow-up were not significantly different between the TFFR approach and the posteromedial (PM) approach [6]. Similar results have also been reported by Jiang et al. at 6 month follow-up [11]. However, the long-term outcomes of the TFFR approach remain unreported.

This study aimed to compare the long-term clinical outcomes of the TFFR and posteromedial approaches for posterior pilon fractures with displaced intercalary fragments over an average 8 year (range 5–12 years) follow-up. We hypothesized that the long-term outcomes of the TFFR approach would not be inferior to those of the classical PM approach.

## Methods

### Study population

This was a retrospective cohort study approved by the Ethics Committee of the authors’ institution and was conducted in accordance with the Helsinki Declaration. We included patients who underwent open reduction and internal fixation surgery by the TFFR approach or the PM approach for posterior pilon fracture from 2012 to 2018. All surgeries were performed by two senior orthopedic trauma surgeons with over 10 years of experience in ankle fracture surgery. The inclusion criteria were as follows: (1) age between 20 and 65 years, (2) closed Klammer classification (classification of fractures involving the tibialis posterior plafond) [13] type 2 (split posterior fragment with potential posteromedial comminution) and type 3 (posterior malleolar fracture line extending anterior to the posterior colliculus, accompanied by a separate anteromedial fragment) posterior pilon fracture with at least one intercalary fragment (Supplementary Fig. S1), (3) the posterior fragment extends along the coronal plane to the posterior colliculus of the medial malleolus on computerized tomography (CT) scan, (4) the intercalary fragment is greater than 5 mm and can be approached through the fibular fracture gap on CT scan, and (5) a distal fibula fracture around the ankle joint (Denis–Weber type B), with the fracture line extending from anterior inferior to posterior superior region. The exclusion criteria were as follows: (1) severe polytrauma or systemic disease (e.g., poorly controlled diabetes, stroke, severe osteoporosis, cardiovascular disease affecting daily activity), (2) no lateral malleolar fracture, (3) lateral malleolar fracture at different planes, and (4) final follow-up at less than 5 years.

## Surgical techniques

### Transfibular fracture region approach

The surgeon places the patient in the unaffected-side lateral position or floppy lateral position [14], depending on the presence of an additional anteromedial medial malleolar fracture, under general or epidural anesthesia, with a tourniquet applied. A longitudinal skin incision is made along the posterolateral border of the fibula, extending sufficiently distally to expose the distal fibula and posterior–lateral aspects of the ankle. The region within 3 cm proximal to and distal to the fibular fracture site is exposed along the anterior aspect of the longus and brevis peroneus muscles. The fracture ends of the fibula are cleaned.

**Fig. 1:**
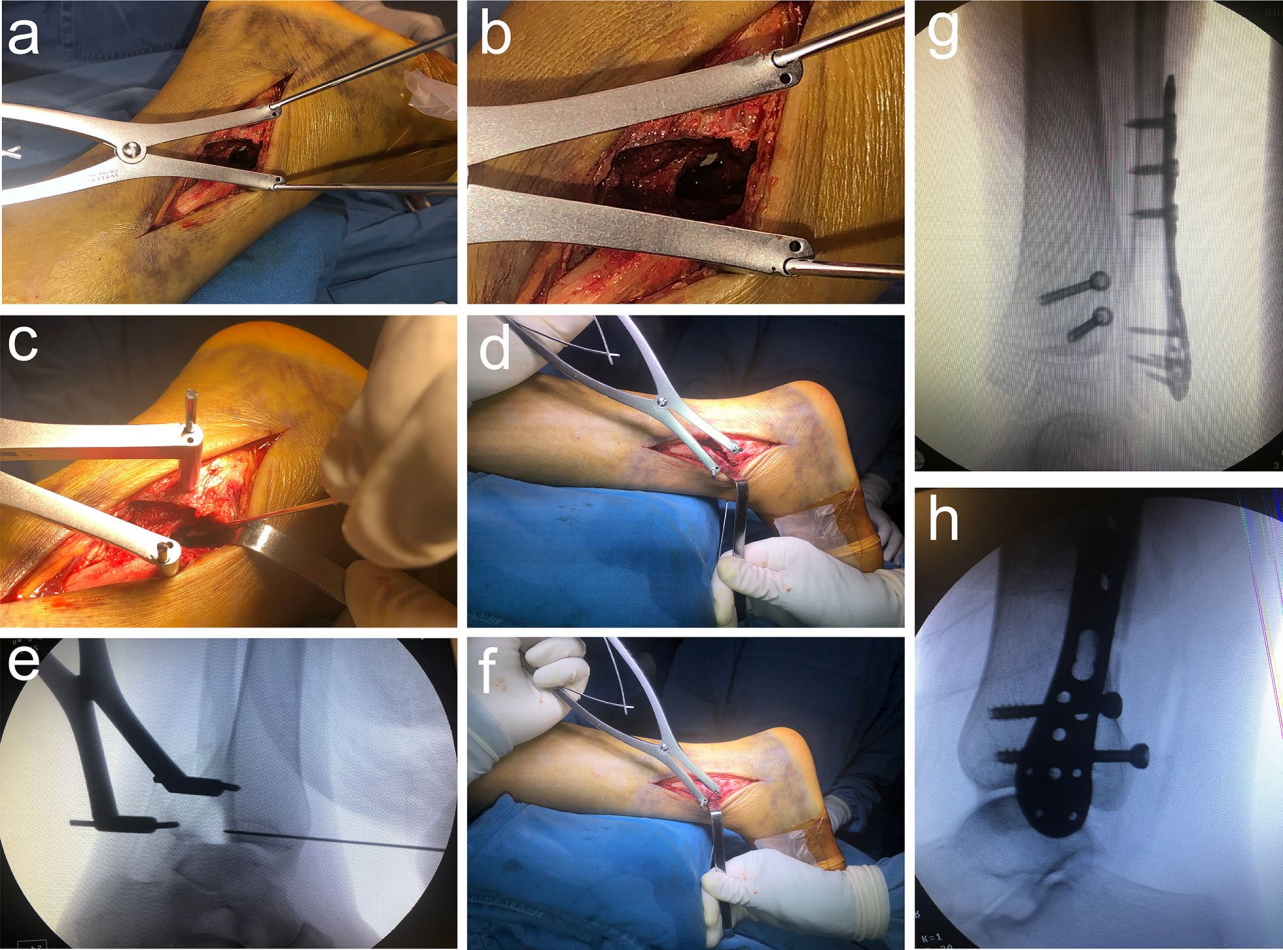
Surgical Procedure

**Fig. 2:**
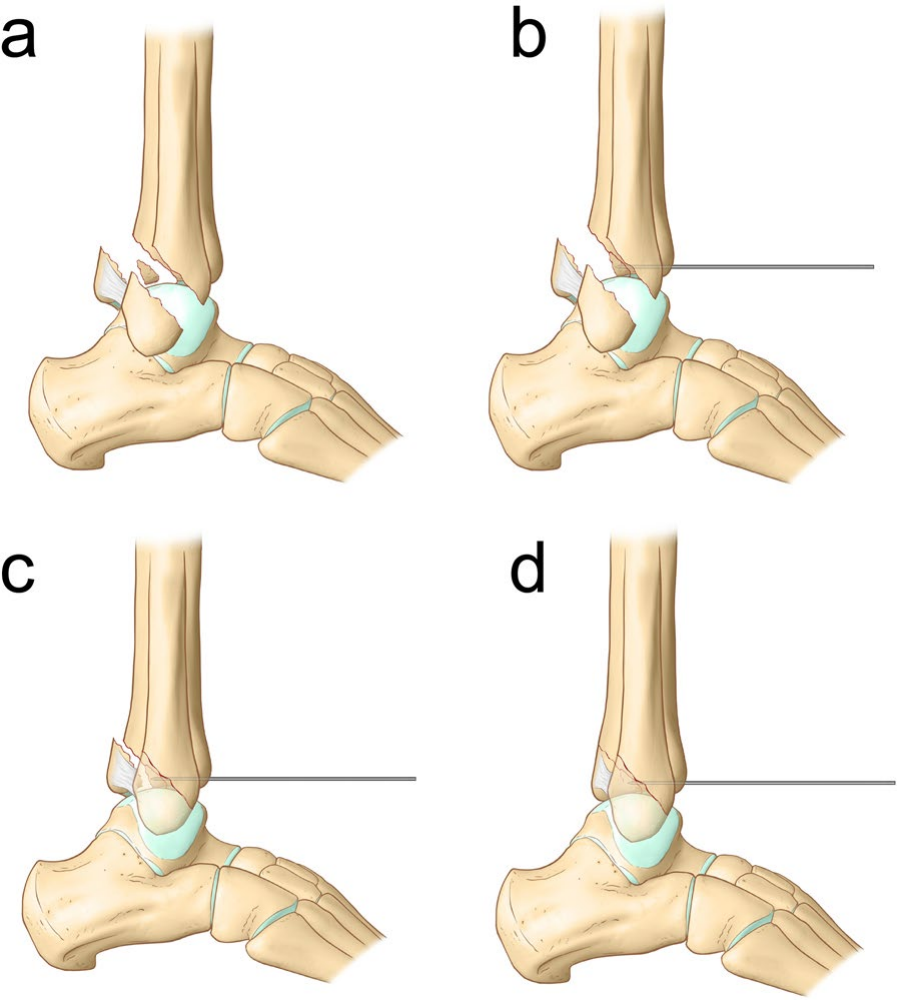
Schematic of surgical procedure

K-wires are placed proximal and distal to the fibula fracture to temporarily stabilize the fracture and allow proper reduction. The distal end of the fibula fracture is pulled backward with a K-wire spreader, to create a gap of approximately 1 cm × 1 cm (Fig. 1a,). The fractured posterior malleolus and impacted joint surface bone fragments are visible under direct view (Figs. 1b, 2a). The intercalary bone fragments are reduced by gently pushing the impacted joint surface back into place with a bone tamp (a tool used to apply gentle force) (Fig. 1c). Temporary fixation of the intercalary joint surface fragments is achieved using an anterior-to-posterior K-wire (Figs. 1d, e, 2b).

The fibular fracture is reduced and fixed with an anatomical or reconstruction plate (Fig. 1f, 2c). The space between the peroneus brevis and flexor hallucis longus muscles is dissected, and care is taken to avoid damaging the sural nerve and small saphenous vein. The flexor hallucis longus muscle is retracted medially. The peroneus longus and brevis muscles are retracted laterally to expose the posterior malleolar fragment. Reduction of the posterior malleolar fracture is achieved by cortical alignment (Fig. 2d). After reduction, buttress plates or screws are used for fixation. If there is a concurrent anteromedial medial malleolar fracture, the patient in the floppy lateral position is then rolled more posteriorly with the operative leg in external rotation. The medial malleolar fracture is reduced through a medial incision and fixed with screws.

After fracture fixation, satisfactory reduction is confirmed using a C-arm X-ray machine under fluoroscopy (Fig. 1 g, h).

### Posteromedial approach

The posteromedial approach utilized in this cohort was originally described by Assal et al., being referred to as the modified posteromedial approach [2]. The patient is placed in the prone position under general or epidural anesthesia, with a tourniquet applied. The knee is flexed and internally rotated. Initially, a posteromedial approach is used. A 6-cm curved incision is made along the posterior medial edge of the Achilles tendon, extending to the medial malleolus. Care is taken to avoid damaging the deep layer of the posterior triangle ligament at the distal end of the incision. The deep fascia is incised, to expose the tibialis posterior retinaculum. The posterior tibial tendon is retracted posteriorly, and soft tissues are dissected along the posterior edge of the tibia. The contents of the tarsal tunnel are retracted posteriorly to expose the posterior malleolus. The posterior fragment is elevated distally, and intercalary articular surface compression fragments are visible. The intercalary fragments are reduced and temporarily fixed in place using K-wires oriented anterior to posterior.

A lateral approach to the lateral malleolus is then performed. The methods used to reduce and fix the lateral malleolar fragment and posterior fragment are the same as those used in the TFFR approach. If there is concurrent medial malleolar fracture, the medial malleolar fragment is reduced through a medial incision and fixed with screws.

### Postoperative management

All patients received standard postoperative management. Passive and active motion of the ankle was encouraged starting from the second day after surgery, as long as pain was tolerable. Patients were allowed to walk with axillary crutches 2 weeks after surgery, but weight bearing was not allowed until 8 weeks post-surgery. Permission to return to work depended on three-dimensional reconstructive CT and clinical signs of healing, usually 12 weeks after surgery.

### Evaluation of clinical outcomes

Postoperative follow-up was conducted through scheduled outpatient visits at 1-, 2-, 3-, 5-, 8-, and 12-month intervals, with subsequent annual remote evaluations. X-ray examination was performed preoperatively; on postoperative day 2; and at the 1-, 2-, 3-, and 12-month follow-up intervals. Computerized tomography (CT) examination with three-dimensional reconstruction was performed preoperatively, immediately post-reduction, and 3 months postoperatively. Clinical outcomes were assessed over an average 8 year follow-up. The surgical duration, intraoperative fluoroscopy times, and postoperative complications were recorded. The time to fracture healing and full weight bearing were observed. Functional outcomes were evaluated via the Foot and Ankle Outcome Score (FAOS), Foot and Ankle Ability Measure (FAAM), and Short Form-36 (SF-36) at last follow-up.

### Statistical analysis

Statistical analysis was performed using the Statistical Package for the Social Sciences 26.0 software (SPSS; IBM, Armonk, NY, USA). Descriptive statistics (means and standard deviations) are used to summarize the data. *p*-Value < 0.05 was considered statistically significant. Normality of the data was assessed using the Shapiro–Wilk test, and differences between groups were compared using Student’s *t* test for continuous variables and the *χ*^2^ test for categorical data.

## Results

**Fig. 3:**
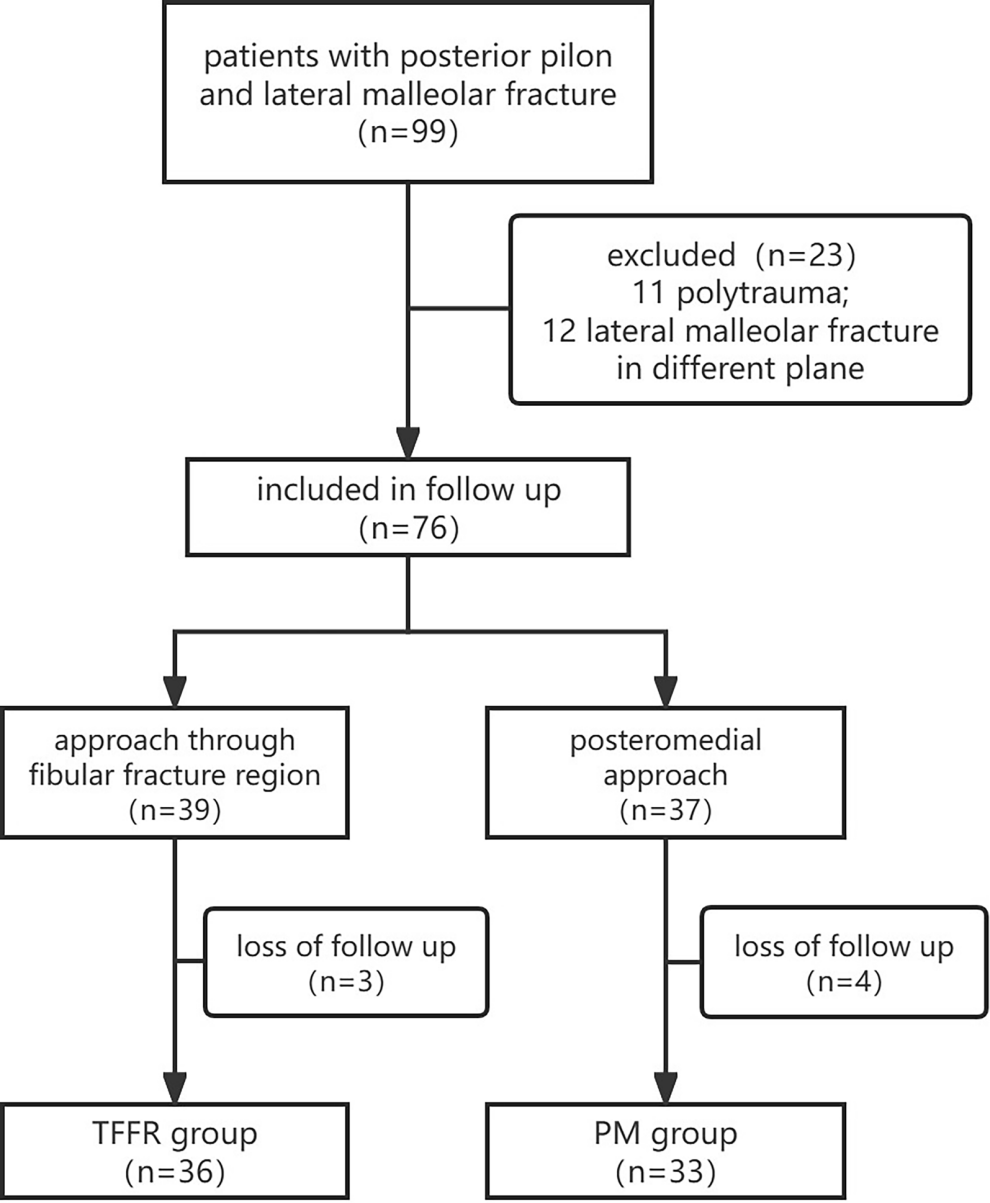
Flowchart

A total of 99 patients with posterior pilon and lateral malleolar fractures who underwent open reduction and internal fixation surgery from 2012 to 2018 met the inclusion criteria. Eleven patients were excluded because of severe polytrauma. Twelve patients were excluded because their lateral malleolar fractures were in a different plane. Seven patients (three in the TFFR group and four in the PM group) were excluded as their last follow-up was less than 5 years owing to loss of contact. Sixty-nine patients completed a minimum 5 year follow-up and were included in the analysis: 36 patients in the TFFR group and 33 patients in the PM group (Fig. 3).

The characteristics of the population are presented in Table 1. No significant differences were found in age, sex, body mass index (BMI-Body Mass Index), smoker status, injury cause, fracture type distribution, or duration from injury to surgery. The average follow-up duration was also similar between the two groups.

**Table 1:** Characteristics of patients

Characteristic	TFFR group (*n* = 36)	PM group (*n* = 33)	*p*-Value
Age, years, mean (SD)	43.5 (8.3)	44.3 (6.9)	0.672
Female, *n* (%)	22 (61.1%)	16 (51.6%)	0.434
BMI, mean (SD)	24.0 (3.1)	24.3 (2.8)	0.685
Smoker, *n* (%)	6 (16.7%)	6 (19.4%)	0.775
Injury causes, *n* (%)			0.442
Falls	29 (80.6%)	24 (72.7%)	
Traffic accident	7 (19.4%)	9 (27.3%)	
Fracture type^ǂ^, n (%)			0.512
Type II	17	13	
Type III	19	20	
Duration from injury to surgery, days, mean (SD)	7.4 (2.8)	7.9 (2.5)	0.447
Follow-up duration, years, mean (SD)	8.3 (2.1)	8.7 (2.0)	0.430

^ǂ^Fracture type classified according to the system developed by Klammer et al.

The clinical outcomes are presented in Table 2. All the fractures healed. After an average 8-year follow-up (range 5–12 years), there was no significant difference between the two groups in FAOS (*p* = 0.679) or its subcomponents: symptoms (*p* = 0.264), pain (*p* = 0.963), activities of daily living (ADL) (*p* = 0.102), sports (*p* = 0.156), and quality of life (*p* = 0.859). No significant differences were found between the two groups in FAAM-ADL (*p* = 0.408), FAAM-Sport (*p* = 0.617), SF-36 physical component score (PCS) (*p* = 0.757), or SF-36 mental component score (MCS) (*p* = 0.594). The time to return to work and intraoperative fluoroscopy were also similar between the two groups. There were two cases of major complications in the TFFR group (looseness of screws) and three in the PM group (one case of looseness of screws and two cases of incision infection). However, no reoperations were needed, as looseness of screws occurred after fracture healing, and the incision infections improved with antibiotic treatment. There were no other major complications, such as joint stiffness, pulmonary embolism, or bone nonunion, in either group. Despite similar long-term outcomes, the surgical duration was significantly shorter in the TFFR group (*p* < 0.001).

**Table 2:** Clinical outcomes

Outcome	TFFR group (*n* = 36)	PM group (*n* = 33)	*p* Value
FAOS, scores, mean (SD)	462.8 (32.9)	459.6 (29.5)	0.679
Symptom	93.8 (3.7)	94.9 (4.3)	0.264
Pain	92.9 (8.1)	92.8 (9.4)	0.963
Daily living	96.7 (2.5)	95.5 (3.4)	0.102
Sports	89.1 (10.1)	85.6 (9.8)	0.156
Quality of life	90.3 (11.4)	90.8 (11.4)	0.859
FAAM, scores, mean (SD)			
ADL	95.1 (5.3)	94.1 (4.4)	0.408
Sports	88.2 (12.1)	86.7 (12.3)	0.617
SF-36, scores, mean (SD)			
PCS	77.5 (15.7)	76.3 (16.6)	0.757
MCS	75.9 (16.7)	73.7 (16.8)	0.594
Complication, *n* (%)	2 (5.6%)	3 (9.1%)	0.572
Looseness of screws	2 (5.6%)	1 (3.0%)	
Infection of incision	0	2 (6.1%)	
Surgery duration, min, mean (SD)	52.4 (9.1)	82.1 (10.8)	< 0.001
Intraoperative fluoroscopy, times, mean (SD)	4.3 (1.0)	4.5 (1.3)	0.474
Return to work time, weeks, mean (SD)	16.8 (2.3)	16.9 (2.4)	0.860

ADL, activities of daily living; PCS, physical component score; MCS, mental component score

**Fig. 4:**
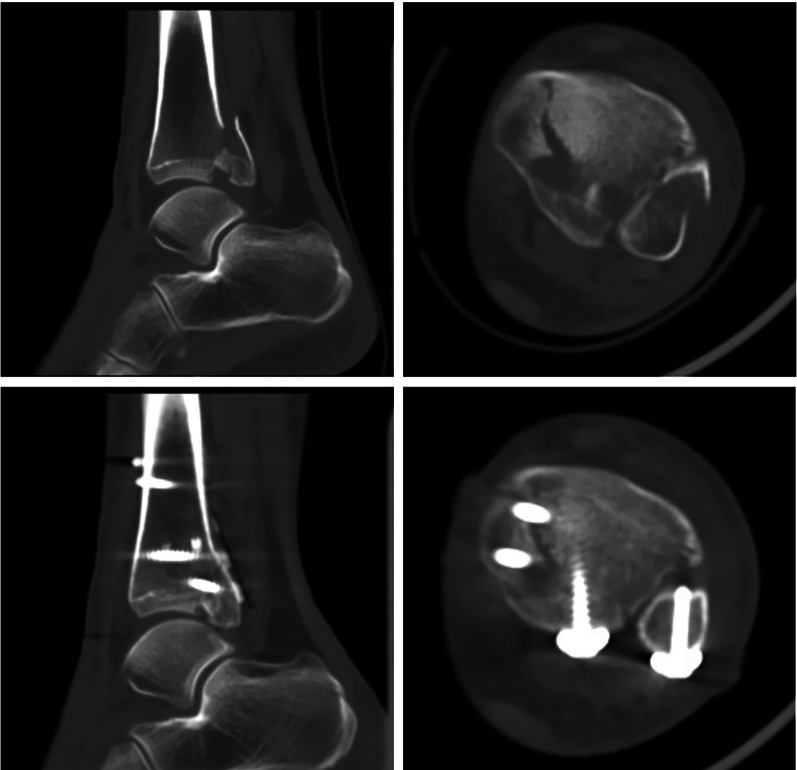
Pre- and postoperative CT images

The radiographic results are presented in Table 3. Fracture reduction was assessed using postoperative CT scans (Fig. 4). The reduction quality was classified into three levels: good (no articular step-off), fair (< 2 mm step-off), and poor (> 2 mm step-off). Posttraumatic arthritis was assessed using the Kellgren and Lawrence arthritis score 1 year after surgery [12]. There were no significant differences in reduction quality or posttraumatic arthritis at 1-year follow up between the two groups.

**Table 3:** Radiographic results

Outcome	TFFR group (*n* = 36)	PM group (*n* = 33)	*p* Value
Reduction^a^			0.831
Good	21	21	
Fair	13	11	
Poor	2	1	
Arthritis^b^			0.347
Stage 0	24	25	
Stage 1	10	8	
Stage 2	2	0	
Stage 3	0	0	

^a^Reduction was evaluated on postoperative CT scan within a week; “good” refers to no articular step-off, “fair” refers to < 2 mm step-off, and “poor” refers to > 2 mm step-off

^b^Arthritis was evaluated on 1 year X-ray by Kellgren and Lawrence arthritis score

## Discussion

The reduction of intercalary fragments, which are crucial for restoring joint function, plays an important role in the recovery of posterior pilon fractures [17]. This study compares the transfibular fracture region approach with the classical posteromedial approach for the reduction of Klammer type 2 and 3 posterior pilon fractures. To our knowledge, there is little data on the long-term outcomes. The most important finding of this study is that the long-term outcomes of the transfibular fracture region approach for reducing intercalary and impacted fragments in posterior pilon fracture with lateral malleolar fractures are not inferior to those of the posteromedial approach. For posterior pilon fractures associated with a lateral malleolar fracture in the same plane and intercalary fragments involving the joint surface, the TFFR approach may be preferred because of its possible decrease in surgical time and use of a single incision.

For posterior pilon fractures, direct visualization of the intercalary fragment and joint surface through the traditional posterolateral approach is difficult because of the obstruction caused by the posterior tibiofibular ligament [13]. Klammer et al. reported an “open book fashion” technique to reveal the joint surface. This technique requires more soft tissue dissection on the posterior side and may still require a posteromedial incision for type 2 and 3 fractures [13]. The fragment of the posterior malleolus in the posterior pilon is usually large and posteromedially positioned. It is difficult to flip the posterior fragment laterally and distally to expose the intercalary fragment because of the Achilles tendon in the rear [18]. However, some posterior pilon fractures are combined with lateral malleolar fractures in the same plane and tend to exhibit posterior–superior to anterior–inferior oblique fracture lines rather than spiral fractures [16]. Haraguchi et al. induced two posterior pilon fractures and five posterior malleolar fractures in a cadaveric study, demonstrating that posterolateral fractures of the tibial plafond were consistently generated by tensile forces transmitted through the posterior inferior tibiofibular ligament [8]. Zhu et al. replicated two posterior pilon fractures in cadaveric models by applying a combined vertical load and external rotation torque with the ankle positioned at 45° plantar flexion and 0° varus [28]. The mechanism of external rotation in plantar flexion with posterior inferior tibiofibular ligament traction may explain the frequent coexistence of posterior pilon fractures and lateral malleolar fractures in the same plane. Specifically, the posterior malleolar fragment is also retracted when the distal end of the fibula is pulled back because of the connection of the posterior tibiofibular ligament. These features provide a basis for the approach through the fibular fracture region. Gonzalez et al. introduced a transfibular approach for posterior malleolar fracture fixation [7]. Chen et al., Liang et al., and Jiang et al. reported promising short-term outcomes of the approach through the fibular fracture region [6, 11, 15]. These studies indicate that fragments and the joint surface can be directly observed through the fibular fracture region. Nevertheless, Liang et al. and Jiang et al. compared the TFFR approach with the posterolateral approach, in which the treatment of the intercalary fragment was not mentioned in detail. Chen et al. reported that the outcomes of the TFFR approach did not differ from those of the posteromedial approach, but at a relatively short-term follow-up.

For posterior pilon fractures, the posterolateral approach has been reported to provide safe exposure for reduction and fixation [5, 13]. However, an additional posteromedial incision is often required to reduce and fix intercalary fragment [5, 13]. The posteromedial approach is appropriate for exposing the posterior malleolar fragment [2]. Bali et al. reported that the posteromedial approach for posterior pilon fracture is a safe technique that enables good visualization and reduction of individual fracture fragments with promising early outcomes [3]. Nevertheless, the posteromedial approach combined with the lateral approach leads to more soft tissue damage and wound complications [26]. In the present study, two surgical-site infections were documented in the PM group. This correlation may be attributed to the cumulative effectiveness of extensive soft tissue dissection and prolonged surgical duration.

Several drawbacks of the TFFR approach should be noted. First, it cannot be performed in all posterior pilon fractures, especially when the lateral malleolar fracture is located in a different plane and intercalary fragments cannot be approached through the fibular fracture gap. The reduction of intercalary fragment located at the posteromedial side may be feasible but challenging, and the posteromedial approach is more comfortable, particularly when a medial incision is needed (Supplementary Fig. S2). Therefore, three-dimensional CT reconstruction is recommended to better understand the fracture pattern involving the posterior malleolar fragment, which aids in surgical planning [17, 22, 23]. Furthermore, the surgeon needs considerable experience and skill for reduction and fixation, as improper reduction of the intercalary fragment may lead to joint incongruence, and redisplacement of the intercalary fragment may cause the formation of loose bodies in the ankle joint. Besides the preoperative CT and the surgical plan, a surgical headlight is recommended to enhance the surgical field of view and ensure the quality of reduction. The ultimate range of the fibular fracture gap that avoids damage to the surrounding ligament structure remains unclear. In our experience, a 1 cm × 1 cm gap is relatively safe and sufficient for exposure. However, further biomechanical studies are needed.

Patients were allowed to return to work as long as bone healing was confirmed. However, they returned to work after approximately 1 month of confirmation. Return-to-work time is strongly affected by subjective and confounding factors, which makes this measure less meaningful in this study.

This study has several limitations. First, this is a retrospective cohort study. We focused on daily function and pain by using scales during the follow-up, but range of motion was not measured. When data concerning complications are collected, inaccuracies in medical records or underreporting by patients could lead to lower rates of complications than were actually experienced by patients. Some patients were lost to long-term follow-up because of lack of prospectively established follow-up plans, and the sample size was relatively small. These factors reduce the strength of our results. Second, long-term fluoroscopy examination was not performed unless the patient complained of pain and dysfunction (Supplementary Fig. S3). Therefore, concurrent radiographic studies could not be performed. Third, all the fractures had healed according to the CT examination, and full weight-bearing was achieved at the 12 week follow-up. As a result, the exact time of fracture healing and full weight bearing was not evaluated and recorded. Fourth, testing fatigue was likely to occur and lead to reduced accuracy as patients were asked to complete the FAOS, FAAM, and SF-36, which contain more than 100 items. Moreover, the BMI ranges in the current study were too low to generalize to obese populations. Further prospective studies with large samples and long-term plans for radiographic monitoring are needed.

## Conclusions

This study retrospectively analyzes the reduction techniques and long-term outcomes of the transfibular fracture region approach or the posteromedial approach for Klammer type 2 and 3 posterior pilon fractures. Both approaches lead to good clinical outcomes, with high function score at an average 8 year follow-up and low arthritis rates at 1 year follow-up. The TFFR approach is not inferior to the posteromedial approach when the intercalary fragment can be accessed through the fibular fracture gap. It may be preferred owing to its potential to reduce surgical time and the use of a single incision.

## Supplementary Information

Supplementary material 1

## Data Availability

The data that support the findings of this study are available from the corresponding author upon reasonable request.
