# Supplementary material for: Reduction and outcome of posterior pilon fractures with intercalary fragments: a retrospective cohort study comparing the transfibular and posteromedial approaches
Source: J Orthop Traumatol. 2025 May 29;26:34. doi: 10.1186/s10195-025-00851-0 (PMC12122404; doi:10.1186/s10195-025-00851-0)
Supplement: Supplementary file 1 — Supplementary material 1 [file 10195_2025_851_MOESM1_ESM.docx]

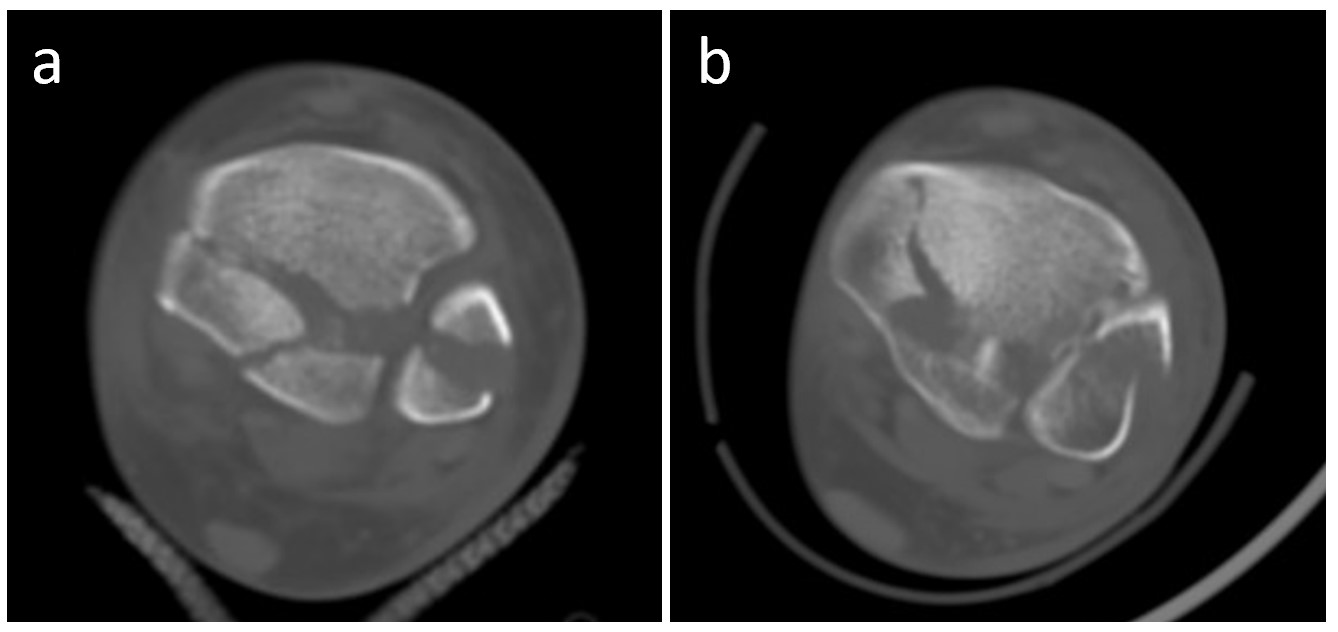


Figure S1. Posterior pilon fracture type. a, Klammer type 2 fracture, posteromedial fracture line may exit medial malleolus through posterior colliculus; b, Klammer type 3 fracture, posteromedial fracture line exits medial malleolus anterior of the posterior colliculus.


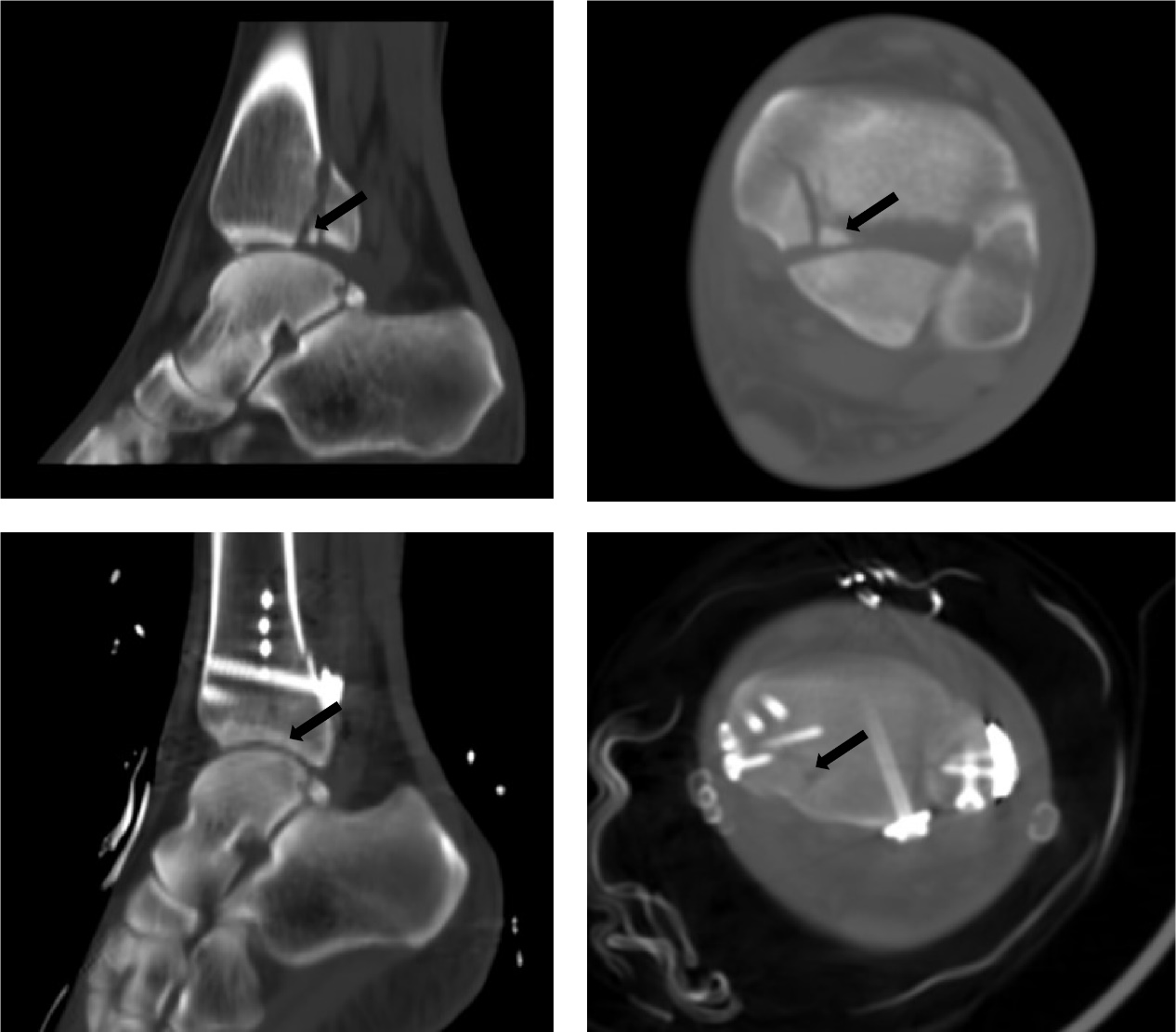


Figure S2. The intercalary fragment (arrow) located at posteromedial side. Although we did reduce the intercalary fragment through fibula fracture region, additional medial incision was indispensable for reduction and fixation of medial malleolar fragment. In this case, potential advantage of single incision was not represented, and posteromedial approach might be more comfortable and easier.


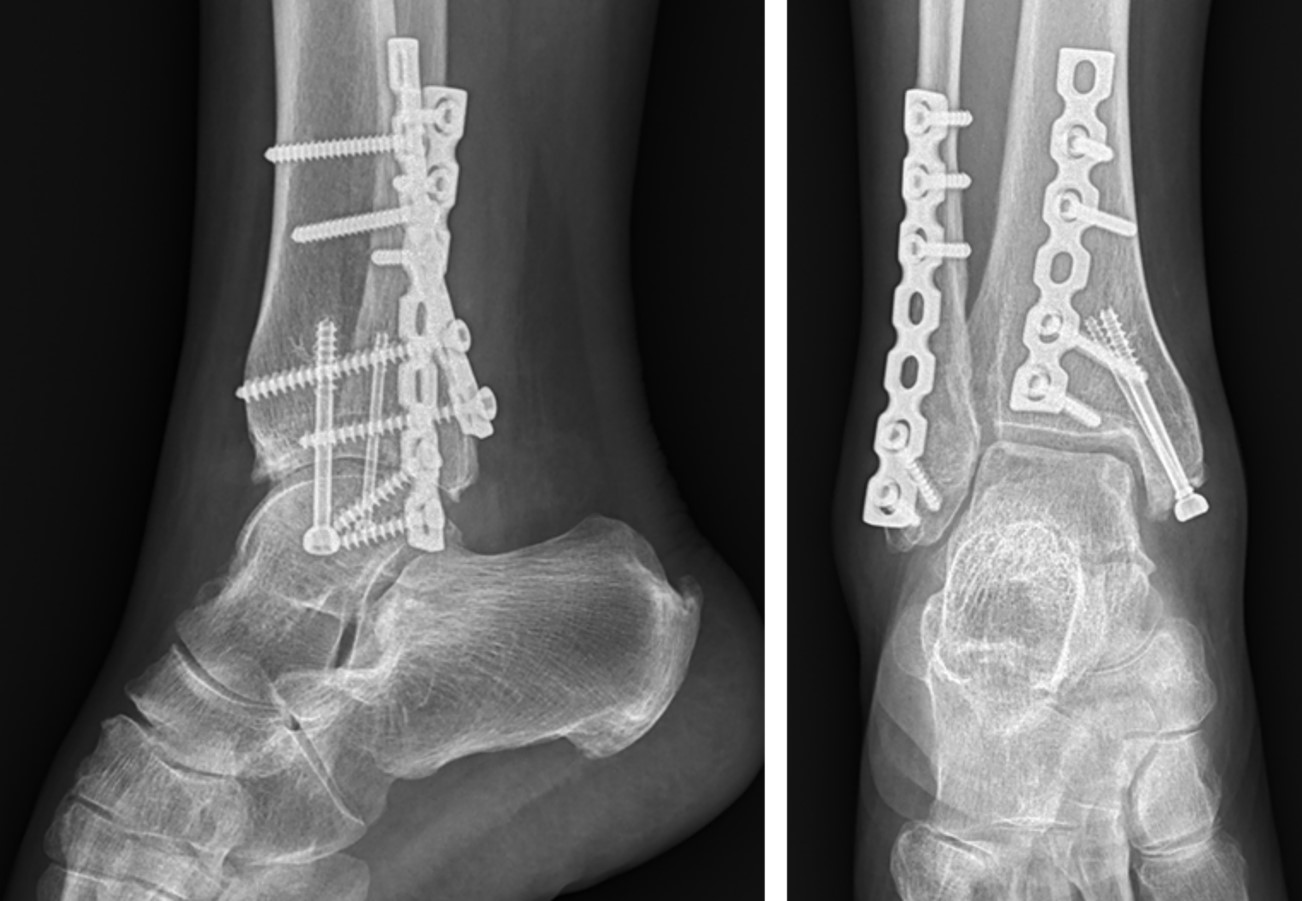


Figure S3. Post-trauma arthritis on fluoroscopy. Fluoroscopy examination was performed two years after surgery as the patient complained about pain and dysfunction.
